# Impact of nutritional screening on mortality and intensive care unit length of stay

**DOI:** 10.3389/fnut.2025.1474039

**Published:** 2025-02-11

**Authors:** Blanca Cecilia Díaz Chavarro, Manuel Romero-Saldaña, Jorge Karim Assis Reveiz, Guillermo Molina-Recio

**Affiliations:** ^1^Nursing Program, School of Health Research Group Genetics, Physiology and Metabolism (GEFIME), Universidad Santiago de Cali, Santiago de Cali, Colombia; ^2^Doctoral Program in Biosciences and Agricultural and Food Sciences, University of Córdoba, Córdoba, Spain; ^3^Nursing, Pharmacology and Physiotherapy Department, University of Córdoba, Córdoba, Spain; ^4^Lifestyles, Innovation and Health (GA—16), Maimonides Biomedical Research Institute of Córdoba (IMIBIC), Córdoba, Spain; ^5^Department of Research and Education, Clínica de Occidente SA, Santiago de Cali, Colombia

**Keywords:** nutritional screening, critical care, malnutrition, mortality, intensive care unit

## Abstract

**Background:**

Nutritional assessment is a fundamental part of the treatment of patients hospitalized in the ICU, allowing the implementation of interventions appropriate to the identified requirements. Since the risk of malnutrition is a modifiable factor, its correct management can positively influence hospital evolution. This study aims to test the impact of the incorporation of nutritional screening and assessment on mortality and length of stay in patients hospitalized in an Intensive Care Unit in Cali, Colombia, during the years 2019 and 2021–2022.

**Methods:**

This is a historical cohort epidemiological study where one cohort consisted of 114 patients who received a standard nutritional screening (interpretation of body mass index and its clinical impression). The other cohort of 630 patients was those exposed to screening with the Malnutrition Universal Screening Tool (MUST) scale. Hematological, clinical, and nutritional variables were considered and their relationship with adverse events, length of hospital stay, and discharge status.

**Results:**

There were significant differences between the two cohorts (*p* < 0.001), with increased mortality and length of hospital stay in patients who received standard nutritional screening without MUST. Furthermore, there was a greater presence of enteral support, diarrhea, anemia, leukocytosis, and lymphopenia in this cohort.

**Conclusion:**

Implementing the MUST screening method and specific nutritional interventions resulted in a significant improvement in patient mortality figures. In addition, the predictive mortality model revealed that emesis and leukopenia increased the probability of death.

## Graphical abstract

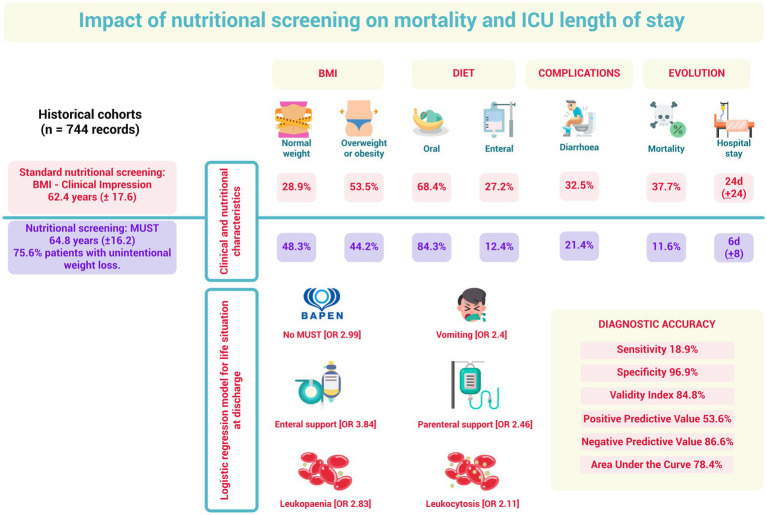

## 1 Introduction

Disease-related malnutrition (DRE) is a complex syndrome resulting from inadequate nutrient intake (1) and the presence of a disease-related systemic inflammatory response (2), including insulin resistance, proinflammatory cytokine activity, and increased release of corticosteroids and catecholamines. This response, coupled with prolonged bed rest, results in a rapid depletion of the body’s energy and nutrient reserves (3). Other factors that may be associated with DRE syndrome include obstruction of the gastrointestinal system, malabsorption of nutrients, misassessment of the patient’s nutritional status, and inadequate administration of nutritional support (4).

From the epidemiological point of view, DRE is of great relevance, both in-hospital and in the community (5). Worldwide, it has been reported that between 30 and 55% of patients admitted to intensive care services are at risk of suffering from malnutrition. However, the percentage varies depending on the population and the criteria used for diagnosis (6). This modifiable risk generates suboptimal results, affecting the hospital course, recovery, and long-term consequences in patients (7), with longer admission time in intensive care units (ICUs) and increased mortality (8).

Therefore, nutritional assessment should be integrated as a fundamental part of the care of hospitalized patients in ICUs and as a general therapeutic strategy during their care (9). The Latin American Federation of Clinical Nutrition and Metabolic Nutritional Therapy, in a study conducted in 2012 in 47 hospitals, highlighted the need for nutritional assessment or screening to be part of the medical records and fulfilled during the first 24–48 h of care. It was reported that only 38% of records had a reference to the nutritional status of hospitalized patients, both in medical and surgical areas and in the ICU, showing a significant underreporting, which leads to a late diagnosis of nutritional risk (10).

A review of the data in Colombia reveals that only 46% of healthcare institutions providing nutrition to patients have an organized nutritional and metabolic support group. Additionally, fewer than 50% of nutritional support groups have the necessary professionals to deliver comprehensive care (11). This shortage is considered a contributing factor to malnutrition, as there is a lack of training and awareness among healthcare professionals and institutions responsible for patient care. Shortages of equipment and supplies and inadequate organizational structures further hinder the provision of care. Consequently, it is crucial to establish systems that facilitate the early identification of malnutrition, address its root causes, monitor nutritional risks, and provide timely and tailored nutritional support for each patient (12).

Moreover, an individualized assessment must be performed for nutritional therapy to be effective, which is not feasible to apply to all patients. Therefore, screening is the starting point to ensure that those who can benefit from nutritional support are identified (13) and, in this way, prevent poor prognosis and mortality associated with malnutrition (14). To carry out these activities, there must be a definition of tasks and responsibilities related to the nutritional care of patients, optimizing communication between the different professionals and hospital levels, and promoting education and continuous training in nutritional knowledge at all levels of care (13). These interventions include the application of objective variables to assess the patient’s condition and body composition, as they allow for the implementation of appropriate strategies to improve the quality of care (15).

At the clinic in Cali, Colombia, where the current investigation took place, it was found that, historically, the ICU did not use a validated scale to identify the risk of malnutrition in its patients. However, starting in 2021, the Malnutrition Universal Screening Tool (MUST) was standardized within the nutritional care protocol. This tool is valid for efficiently identifying nutritional risk in a specific patient population, is easy to use, and contains direct and objective questions, which facilitates its use in time-limited settings (16). Thus, it was possible to classify the level of nutritional risk, the type of nutritional support required for each patient and follow-up, the frequency of reassessment, and the specialist in charge of follow-up. Therefore, this study aimed to test the impact of incorporating nutritional screening and assessment of mortality and length of stay in patients hospitalized in an Intensive Care Unit in Cali, Colombia, during 2019 and 2021–2022.

## 2 Methods

### 2.1 Design, population, and sample

A historical cohort epidemiological study was carried out. One cohort consisted of patients seen in the ICU during 2019 who received standard nutritional screening performed by the nutrition and dietetics team through the interpretation of body mass index (BMI) and their clinical impression. The other cohort of patients were those exposed to nutritional screening with the MUST scale, seen in the ICU from 2021 to 2022.

**Figure 1:**
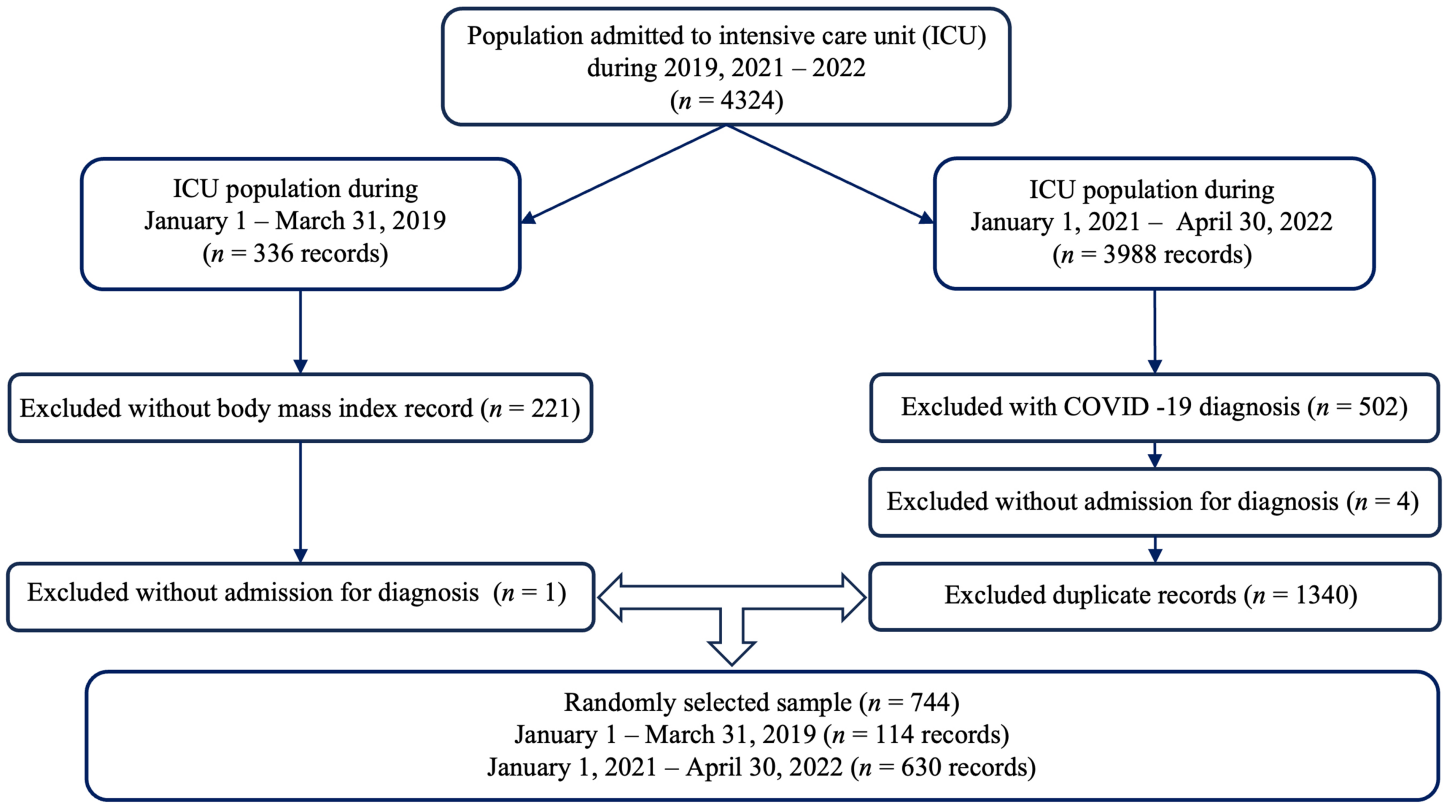
Diagram of sample selection.

The total number of patients admitted to the adult ICU service during the study period was 4,324. The epidemiological statistical package Epidat version 4.2 was used to calculate the sample size. With a risk of DRE of 80% for patients receiving routine health care (17–19) vs. a 29.6% probability of DRE in patients undergoing nutritional screening (19), a ratio between groups equal to 1, a loss rate of 10%, a safety of 95%, a power of 90%, and applying a Yates continuity correction, a total sample of 52 patients was obtained, 26 patients per group. In any case, to ensure homogeneity in the sample, data were collected from 114 patients seen during 2019 (usual nutritional care) and from 630 patients seen during the second half of 2021 and the first half of 2022 (MUST nutritional screening), which allowed for a comparative analysis between the two cohorts, with a total of 744 patient records (Figure 1).

### 2.2 Selection criteria

#### 2.2.1 Inclusion criteria

The study’s inclusion criteria were as follows: patients seen within the ICU during 2019 to whom the MUST scale was not applied; patients admitted to the adult ICU service during the second semester of 2021 and the first semester of 2022 to whom the MUST scale was applied; and patients ˃18 years.

#### 2.2.2 Exclusion criteria

Patients hospitalized for COVID-19 were excluded. In addition, data from patients hospitalized during the year 2020 were omitted since a significant percentage of admissions was caused by this same pathology. This decision was made because, in this type of patient, there are some differentiating factors (reduced food intake, catabolism related to inflammation, decreased appetite, diarrhea, longer length of hospital stay, etc.) (20), which could bias the comparative results.

### 2.3 Variables and measurement

#### 2.3.1 Explanatory variables

The following sociodemographic variables were taken into account: sex, age, and life course classification according to Resolution No. 3280 of Colombia, which identifies young people between 18 and 28 years, adults between 29 and 59 years, and older adults, those aged ≥60 years (21), and the disease of entry according to the International Classification of Diseases 11. Hematologic variables included hemoglobin levels, hematocrit percentage, leukocytes, and lymphocytes. Within the clinical variables, the presence of gastrointestinal symptoms such as hyporexia, bloating, abdominal pain, diarrhea, dysphagia, and emesis was included. Finally, nutritional variables were considered, such as the type of support received (oral feeding, enteral support, parenteral support, or mixed support) and the nutritional assessment according to MUST, which identified patients with low, medium, and high nutritional risk.

#### 2.3.2 Resulting variables

The following were considered as outcome variables: days of hospitalization, adverse events such as skin lesions associated with care dependency and healthcare-related infections (surgical site infection, urinary tract infection, pneumonia), and patient discharge status (alive or deceased).

### 2.4 Measurement tools

#### 2.4.1 Standard nutritional assessment

The BMI, calculated as kg/m^2^, and the subjective clinical impression of nutritional status (carried out by the hospital nutrition team) were taken into account, classifying patients with a BMI <18.5 kg/m^2^ as underweight patients, BMI 18.5–24.9 kg/m^2^ as normal weight, BMI 25.0–29.9 kg/m^2^ as overweight, and BMI ≥30.0 kg/m^2^ as obese (22).

#### 2.4.2 Nutritional assessment with MUST

The MUST scale takes into account the analysis of three scores: (a) BMI, which is scored ≥20 kg/m^2^ = 0; 18.5–20 kg/m^2^ = 1; and ≤ 18.5 kg/m^2^ = 2; (b) unintentional weight loss during the past 3–6 months, calculated as a percentage and coded as follows: ≤5% = 0; 5–10% = 1; and ≥ 10% = 2; and (c) the effect of acute illness, where 2 points are assigned when there has been or is likely to be no nutritional intake for >5 days. The cumulative value of these three scores classified patients into three risk levels: 0 points = low risk; 1 point = medium risk; and ≥ 2 points = high risk (23, 24).

#### 2.4.3 Assessment of nutritional needs and care

Those responsible for the evaluation of the nutritional status of the patients were the nutritionists of the health institution in response to the request of the ICU medical team. The frequency of the assessment depended on the level of risk identified in each patient. For low-risk patients, it was determined that the assigned physician would establish the dietary guidelines, and the screening was repeated weekly. For medium-risk patients, dietary intake was monitored for three consecutive days, and if sufficient, rescreening was performed weekly. For patients at high nutritional risk, follow-up was performed by the nutrition and dietetics unit, which established a treatment protocol and evaluation of the nutritional care plan.

Gastrointestinal symptoms were assessed by both the medical staff and the nutrition and dietetics team during the patient’s admission. This information was obtained through anamnesis with verbal reference from the patient (if the patient’s clinical condition permitted) or through information provided by the patient’s primary caregiver. This information was complemented with data from the physical assessment performed by the physician responsible for the patient in the ICU. In the case of hyporexia, it was documented from the verbal reference of the patient’s decreased appetite and oral intake.

The nutritional support collected in the study corresponds to that indicated in the patient’s clinical history, at the time of consultation with the nutrition and dietetics team. In this study, changes in the type of nutritional support were not recorded, considering that only the data from the first nutritional screening were collected, together with the results of the paraclinical tests on admission; analyzing the patient’s condition at a single moment of hospitalization, so that all the data would be related to the nutritional risk identified.

### 2.5 Ethical aspects

This research adhered to the international postulates for health-related research on human subjects, created by the Council for International Organizations of Medical Sciences in collaboration with the World Health Organization and the Declaration of Helsinki for the participation of human subjects in research. Approval was received from the Ethics and Bioethics Committee of the Department of Health of the University Santiago de Cali-Colombia—"CEB-USC,” in the session held on June 26, 2020, according to Minutes N° 01 and by the Scientific Technical Committee of the participating clinic, under registration IYECDO-1358 of February 11, 2021.

### 2.6 Statistical analysis

Statistical analysis was performed with SPSS software version 28.0. The characterization of sociodemographic factors such as life course, sex, and clinical factors such as the pathology causing hospitalization of the patients admitted to the ICU was performed by means of absolute and relative frequency tables. In addition, weight loss, global risk of malnutrition, and percentage of weight loss were determined, according to data reported in patients with MUST scale assessment.

Absolute and relative frequency analyses were performed on the nutritional factors and the interpretation of the patients’ paraclinical results, comparing the standard nutritional care cohort (2019) vs. the one seen after the implementation of the MUST scale (2021–2022), evaluating their association by means of Pearson’s chi-square test, with their respective corrections, when necessary. For the paraclinical data analyzed, the Kolmogorov–Smirnov test with Lilliefors correction was applied, finding that there was no normal distribution, so the comparison of means was performed through the Mann–Whitney U test.

A binary logistic regression model was performed for the mortality outcome, determining the adjusted OR values and the Hosmer and Lemeshow test for the goodness-of-fit of the model, as well as the Wald statistic and the Cox and Snell and Nagelkerke R^2^ coefficients of determination. In all cases, a statistical significance level of *p* < 0.05 was considered.

## 3 Results

### 3.1 Sociodemographic characterization of ICU patients

The sample of 744 admissions to the Intensive Care Unit in Cali, Colombia, was divided into two cohorts. The first corresponds to a standard nutritional program equivalent to 15.3% of care provided during 2019, while the rest corresponds to a nutritional program that included the MUST scale in patients seen between June 2021 and June 2022.

The diagnosis of circulatory and respiratory system pathologies was the most prevalent in both cohorts, but there were statistically significant differences (*p* < 0.001) related to a higher percentage of these pathologies during the 2021–2022 cohort (50% vs. 37.3%) and a higher percentage of neoplasms in patients seen during 2019 (29.8% vs. 17%). Significant associations or differences (*p* < 0.001) were also identified, with a mortality of 37.72% in patients seen during 2019 vs. a mortality of 11.6% in patients seen during 2021–2022 and a higher average number of days of hospital stay in patients who did not have MUST scale assessment performed within their nutritional care (24 vs. 6 days) (Table 1).

**Table 1:** Sociodemographic characteristics and progress of patients admitted to the intensive care unit (ICU) of the Cali, Colombia clinic during 2019 vs. 2021–2022.

Sociodemographic characteristics		Cohort				*p*
		2019 (without nutritional screening)		2021–2022 (with nutritional screening)		
		*n* / $\bar{x}$	% / (±SD)	*n* / $\bar{x}$	% / (±SD)	
Age (years)		62.4	17.6	64.8	16.2	0.156
Life course	Youth (18–28 years old)	9	7.9%	22	3.5%	0.038*
	Adulthood (29–59 years)	38	33.3%	169	26.8%	
	Old age (≥60 years)	67	58.8%	439	69.7%	
Sex	Male	48	42.1%	341	54.1%	0.018*
	Female	66	57.9%	289	45.9%	
International Classification of Diseases 11 grouping pathologies	Circulatory and respiratory systems	43	37.7%	315	50.0%	<0.001*
	Neoplasms	34	29.8%	107	17.0%	
	Infectious and parasitic	13	11.4%	33	5.2%	
	Endocrine and digestive systems	12	10.5%	47	7.5%	
	Nervous system and trauma	9	7.9%	83	13.2%	
	Other diseases	3	2.6%	45	7.1%	
Progress of patients admitted to the ICU						
Presence of adverse event	No	91	79.82%	528	83.8%	0.295
	Yes	23	20.18%	102	16.2%	
Vital situation at discharge	Survival	71	62.28%	557	88.4%	<0.001*
	Mortality	43	37.72%	73	11.6%	
Days of hospital stay		24	24	6	8	<0.001*

Adverse event: The result of an unintended harm generated during the care process of a disease, which has a negative effect on the patient’s health (50). n, number; $\bar{x\;}$, average; %, percentage; SD, Standard Deviation.

The incidence rate (IR) of mortality in the 2019 cohort was 15.7 deaths per 1,000 ICU patients/day, while the IR of adverse events was 8.4 cases per 1,000 ICU patients/day. For the 2021–2022 cohort, the mortality IR was 19.1 deaths per 1,000 ICU patients/day, and the adverse event IR was 26.7 cases per 1,000 ICU patients/day.

### 3.2 Nutritional risk assessment according to the MUST scale

In the cohort seen between 2021 and 2022, it was found that 3 out of 4 patients had reported unintentional weight loss during the last 6 months, with an average loss of 4.85% of body weight (standard deviation [SD] 6.20%). In determining nutritional risk according to the MUST scale, a high risk of malnutrition was reported in 28.4% (Table 2).

**Table 2:** Malnutrition universal screening tool (MUST) scale characteristics of patients admitted to the intensive care unit of the Cali, Colombia clinic during 2021–2022.

Must scale		*n* / $\bar{x}$	% / (±SD)	CI 95%	
				Lower	Upper
Presence of weight loss	No	154	24.4%	21.2%	27.9%
	Yes	476	75.6%	72.1%	78.8%
Unintentional weight loss last 6 months (%)		4.85	6.20	4.37	5.34
Global risk of malnutrition categorized MUST	Low	383	60.8%	56.9%	64.5%
	Medium	68	10.8%	8.6%	13.4%
	Hight	179	28.4%	25.0%	32.0%

n, number; $\bar{x\;}$, average; %, percentage; SD, Standard Deviation; CI, Confidence Interval.

### 3.3 Assessment of clinical, nutritional, and hematological characteristics

When analyzing BMI, there were statistically significant differences (*p* < 0.001) showing a higher percentage of patients with normal BMI in the 2021–2022 cohort (48.3% vs. 28.9%). Regarding diet, a higher use of enteral support was found in the 2019 cohort and a higher use of the oral route in patients seen between 2021 and 2022. When looking at the results related to nutritional supplementation through the addition of protein modules in the diet of patients, there were no significant statistical differences between cohorts.

For gastrointestinal symptoms, a greater presence of diarrhea was identified in the 2019 cohort, with a decrease of 11% of patients with this symptom during 2021–2022 admissions (*p* = 0.010). Within the alterations presented in the hematological parameters of the two cohorts, anemia was more prevalent in patients hospitalized during 2019 (74.6% vs. 62.1%; *p* = 0.024), and the same occurred with leukocytosis (47.4% vs. 27.9%; *p* < 0.001) and with lymphopenia (64.9% vs. 49.8%; *p* = 0.012; Table 3).

**Table 3:** Clinical, nutritional, and hematological characteristics of patients admitted to the intensive care unit (ICU) of the Cali, Colombia clinic during 2019 vs. 2021–2022.

Clinical and nutritional factors		Cohort				*p*
		2019		2021–2022		
		*n*	%	*n*	%	
Body Mass Index - BMI	Low weight	20	17.5%	48	7.6%	<0.001*
	Normal	33	28.9%	304	48.3%	
	Overweight	35	30.7%	192	30.5%	
	Obesity	26	22.8%	86	13.7%	
Diet	Oral route	78	68.4%	531	84.3%	<0.001*
	Enteral support	31	27.2%	78	12.4%	
	Parenteral support	4	3.5%	21	3.3%	
	Mixed support	1	0.9%	0	0	
Addition of protein module	No	82	71.9%	423	67.1%	0.314
	Yes	32	28.1%	207	32.9%	
Hyporexia	No	86	75.4%	494	78.4%	0.481
	Yes	28	24.6%	136	21.6%	
Abdominal distention	No	73	64.0%	428	67.9%	0.414
	Yes	41	36.0%	202	32.1%	
Diarrhea	No	77	67.5%	495	78.6%	0.010*
	Yes	37	32.5%	135	21.4%	
Abdominal pain	No	67	58.8%	393	62.4%	0.465
	Yes	47	41.2%	237	37.6%	
Nausea	No	73	64.0%	415	65.9%	0.704
	Yes	41	36.0%	215	34.1%	
Dysphagia	No	88	77.2%	477	75.7%	0.734
	Yes	26	22.8%	153	24.3%	
Emesis	No	68	59.6%	412	65.4%	0.238
	Yes	46	40.4%	218	34.6%	
Constipation	No	87	76.3%	424	67.3%	0.056
	Yes	27	23.7%	206	32.7%	
Hemoglobin	Polycythemia	4	3.5%	54	8.6%	0.024*
	Normal range	25	21.9%	185	29.4%	
	Anemia	85	74.6%	391	62.1%	
Leukocytes	Leukocytosis	54	47.4%	176	27.9%	<0.001*
	Normal range	46	40.4%	400	63.5%	
	Leukopenia	14	12.3%	54	8.6%	
Lymphocytes	Lymphocytosis	3	2.6%	19	3.0%	0.012*
	Normal range	37	32.5%	297	47.1%	
	Lymphopenia	74	64.9%	314	49.8%	

n, number; %, percentage; BMI, Body Mass Index. The symbol * means *p* < 0.05.

### 3.4 Multivariate analysis for the vital situation at discharge of ICU patients

In the logistic regression model for the variable “vital status at discharge,” the following predictor variables were significant: the cohort in which the patients were admitted, the type of nutritional support received, the presence of emesis, and the leukocyte levels.

In patients admitted during the 2019 period (without application of MUST screening), a nearly 3-fold increased risk for mortality was found, while in those who required parenteral nutritional support, this risk increased 2.46-fold vs. those who received oral feeding. Similarly, emesis during hospitalization and leukopenia were associated with an increased likelihood of death (2.40 [1.528–3.769] and 2.83 [1.434–5.570], respectively; Table 4).

**Table 4:** Multivariate logistic regression model for vital status at discharge according to cohort.

Raw estimate (unadjusted)						Adjusted estimate		
Variables	Living *n* (%)	Deceased *n (%)*	OR	CI 95%	*p*	OR	CI 95%	*p*
Cohort								
With MUST screening	557 (88.41%)	73 (11.59%)	1 (Ref.)	–	–	1 (Ref.)	–	–
No screening	71 (62.28%)	43 (37.72%)	4.621	2.945–7.251	<0.001	2.990	1.806–4.948	0.000
Nutritional support								
Oral route	531 (90%)	59 (10%)	1 (Ref.)	–	–	1 (Ref.)	–	–
Enteral support	74 (62.71%)	44 (37.29%)	5.351	3.378–8.477	<0.001	3.837	2.337–6.301	0.000
Mixed support	1 (100%)	0	0.000	0.000	1.000	0.000	0.000	1.000
Parenteral support	22 (62.86%)	13 (37.14)	5.318	2.546–11.108	<0.001	2.458	1.088–5.551	0.031
Emesis								
No	429 (89.38%)	51 (10.63%)	1 (Ref.)	–	–	1 (Ref.)	–	–
Yes	199 (75.38%)	65 (24.62%)	2.748	1.836–4.113	<0.001	2.400	1.528–3.769	0.000
Interpretation of leukocyte levels								
Normal range	403 (90.36%)	43 (9.64%)	1 (Ref.)	–	–	1 (Ref.)	–	–
Leukopenia	50 (73.53%)	18 (26.47%)	3.374	1.808–6.296	<0.001	2.826	1.434–5.570	0.003
Leukocytosis	175 (76.09%)	55 (23.91%)	2.946	1.903–4.558	<0.001	2.106	1.312–3.380	0.002

When constructing an explanatory model of mortality with the final variables that remained after the adjusted estimation of the odds ratios (ORs), the omnibus test was significant (*p* < 0.001), and a goodness-of-fit of 0.957 was obtained, with a high specificity of 96.97%, an area under the curve (AUC) of 78.4%, and a validity index of 84.81% (Table 5).

**Table 5:** Diagnostic accuracy for vital status at discharge.

	Reference test		
Diagnostic test	Mortality	Survival	Total
Positive	22	19	41
Negative	94	609	703
Total	116	628	744
	Value	CI (95%)	
Sensibility (%)	18.97	11.40	26.53
Specificity (%)	96.97	95.56	98.39
Validity rate (%)	84.81	82.17	87.46
Predictive value (+) (%)	53.66	37.18	70.14
Predictive value (−) (%)	86.63	84.04	89.22
Prevalence (%)	15.59	12.92	18.27
Youden Index	0.16	0.09	0.23
Likelihood ratio (+)	6.27	3.51	11.21
Likelihood ratio (−)	0.84	0.76	0.91
Area under the curve	0.784	0.738	0.831

Omnibus tests of model coefficients: <0.001. Cox and Snell R-squared: 0.137. Nagelkerke’s R-squared: 0.237. Hosmer and Lemeshow test: 0.957.

## 4 Discussion

Data from 744 patients hospitalized in the ICU of a clinic in Cali were analyzed and divided into two cohorts according to the method of nutritional assessment used: BMI or application of the MUST scale.

### 4.1 Sociodemographic characterization and progress of ICU patients

Regarding the sociodemographic characterization, it was determined that the group of older adults (>60 years) had a greater proportion in the 2021–2022 cohort. In view of this data, the literature indicates that there is limited information regarding the clinical outcomes of older adults admitted to the ICU. This occurs even though their admissions have increased worldwide (25), generally due to causes related to the presence of chronic diseases (26), such as circulatory pathologies and neoplasms, which increase their incidence with increasing age (27). This aspect was evidenced in the results of our study, where these were the most frequent pathologies in both cohorts.

Regarding the progress of people admitted to the ICU, a study with data from Korean patients seen from 2009 to 2014 found that the average length of hospital stay was 4 days, and the overall mortality was 13.8 (28). However, in data from ICUs from 45 countries, a mortality of 17.12% (confidence interval: 16.93–17.32) was found in patients without healthcare-associated infections, with an average length of stay in the ICU of 8.07 (8.01–8.10) days (29).

These data reflect that there is an important variation in the progress of patients hospitalized in the ICU, which can be affected by variables such as pathologies causing the admission, the inflammatory state manifested during hospitalization, the presence of adverse events such as infections associated with health care, the immunological response, and the nutritional status of patients. It is important to highlight here the role of nutritional risk screening performed with the population of patients seen during 2021–2022 in Cali, which allowed for rapid identification and prioritization of clinical interventions, which in the literature has been shown to have a positive impact on aspects such as length of stay in the ICU, morbidity, and mortality (30). This fact was also demonstrated in the results of this research.

### 4.2 Nutritional risk assessment according to MUST

When reviewing international data on the use of the MUST scale, results were found from a hospital in Australia with patients admitted to the ICU, where 20% of patients were identified as having a high nutritional risk, and 15% presented a medium risk (31). Similar proportions were observed in the cohort seen in the Cali ICU between 2021 and 2022, where 28.4% of patients had a high risk of malnutrition and 10.8% presented a medium risk.

Furthermore, in this same cohort of patients, it was found that three out of four had experienced unintentional weight loss during the last 6 months, with an average of 4.85% of their body weight (SD: 6.20%). This variable is part of the data collected in the application of the MUST scale (32), as weight loss greater than 5% in a short period has been associated with a deterioration in nutritional status (33). Therefore, some healthcare centers have used this data to evaluate their patients. A multivariate analysis performed on patient data from two hospitals in Toronto, Canada found that subjective global assessment ratings of nutritional status were significantly affected by weight loss (34).

### 4.3 Assessment of clinical, nutritional, and hematological characteristics

A study conducted in Australia evaluated the information collected by dietitians from subjects admitted to the ICU, including the type of nutritional support received and the symptoms that affected the patients’ nutrition. The study found that oral feeding was the most common form of nutritional support, with 80% of patients receiving it (35). This finding is similar to that obtained with the data from the 2021–2022 cohort in Cali, where 84.3% of patients received oral feeding. In contrast, the 2019 cohort had a higher utilization of enteral support.

Differences in the type of nutritional support provided could be influenced by the presence of gastrointestinal symptoms. However, in our study, no statistically significant differences were observed between the two cohorts that could account for this variation. Both cohorts exhibited a high frequency of abdominal pain, followed by emesis and, to a lesser extent, hyporexia. It is important to consider that several relevant factors determine the route of nutritional support, including the patient’s ability to eat safely and adequately, the nutritional goals, the risk of aspiration, the functional status of the gastrointestinal tract, cognitive function and skills, availability of enteral and/or vascular access, and the results of tests and invasive procedures performed in the ICU (36).

The 2019 patient cohort exhibited a higher inflammatory status, evidenced by a greater prevalence of anemia and leukocytosis, likely related to the underlying disease prompting ICU admission. This higher inflammatory status may have resulted in increased utilization of alternative nutritional support routes, thereby overcoming barriers to oral intake and adhering to general recommendations for adjusting nutritional therapy based on the patient’s clinical condition regarding safety and efficacy (36).

Moreover, the hematological status of the patients also allowed us to identify a high frequency of lymphopenia in the 2019 population, this being a characteristic of immunosuppression, which is usually present on admission to the ICU (37, 38), regardless of whether or not there is a diagnosis of sepsis, thus being able to generate a poor prognosis. Other studies have supported its relevance as a predictive biomarker and possible therapeutic target in intensive care medicine (39). The high presence of lymphopenia in this cohort could have had an impact on patient discharge outcomes. However, the lack of follow-up data on these patients when transferred to other hospital services prevents us from confirming this fact (39).

Regarding supplementation with protein modules for Cali ICU patients, a higher percentage of this type of dietary therapeutic intervention was identified in the 2021–2022 cohort, although without presenting statistically significant differences with the 2019 cohort. In the study by Amon et al., it was observed that a diet high in energy and protein was the most common code assigned, with a mean cumulative nutritional adequacy of 47% (30–74%) for protein, presenting a high percentage of supplementation of this nutrient. This type of intervention has been associated with better clinical outcomes (40). In our study, introducing MUST screening in the care protocol could have been beneficial because it could have led to more precise interventions according to the risk identified in the population served during 2021–2022.

### 4.4 Multivariable model for vital situation at discharge

Estimating nutritional risk is often not considered in clinical practice (41), even though it has been shown that such detection and early treatment of malnutrition reduce morbidity and mortality and improve patient outcomes (42). In the case of the cohort admitted to the Cali ICU during the 2019 period (without application of the MUST scale), this aspect was evidenced, identifying a risk increased by almost 3 times for mortality during hospitalization. For these reasons, the need for adequate nutritional screening and assessment tools is evident, as they will help to identify effective strategies to reduce the negative impact of malnutrition (30).

Conversely, in our study, it was observed that patients who received enteral and parenteral nutritional support presented a higher risk of mortality compared to those who were fed orally. This type of support is recommended for critically ill patients with malnutrition who are unable to feed themselves due to their clinical conditions. It is possible that the greater severity of their condition, requiring the use of enteral or parenteral nutrition, contributes to the higher mortality rate in these patients. The literature is inconclusive regarding the comparison between tube feeding and oral diet with intravenous dextrose (standard care) vs. parenteral nutrition. Some studies suggest that tube feeding and standard care are associated with a lower infection risk than parenteral nutrition. However, mortality and risk of infection appear to be higher with standard care in malnourished populations (43).

It is important to mention that, according to the 2019 European Society for Clinical Nutrition and Metabolism recommendations, to avoid overfeeding patients, enteral and parenteral nutrition should be prescribed gradually between 3 and 7 days, avoiding excessive nutrient intake at the beginning of hospitalization. In addition, it is recommended that parenteral support be indicated only when all strategies to promote tolerance to enteral nutrition have been maximized, and this goal has not been achieved (44).

Food intolerance may present with gastrointestinal symptoms, such as emesis, high gastric residual volume, absence of gastrointestinal peristalsis, abdominal distension, and diarrhea (45). These symptoms have also been associated with an increased risk of mortality, generating an OR of up to 5.24 in surgical ICU patients (46). In our study, we observed that emesis increased the risk of mortality 2.4-fold. Therefore, it is essential to perform a thorough evaluation to identify food intolerance and provide an adequate approach to the patient, focusing on recovery of health status and reducing complications.

Regarding the changes in the levels of white blood cells present in our Cali patients and their relationship with the increase in mortality, it has been shown that these data are clinically significant and valuable for diagnosing and controlling the condition of hospitalized people (47). These findings coincide with a meta-analysis, demonstrating a clinically significant relationship between high white blood cell count and mortality in various study populations (47, 48).

Finally, an investigation in Greece with critically ill patients found that the Acute Physiology and Chronic Health Evaluation II classification system scale correlated well with in-hospital mortality, showing an AUC of 0.6684. This result suggests moderate discrimination in a mixed ICU population (49), similar to that of the population in our study. However, this research developed a predictive model for mortality with a higher AUC (0.784), which underlines the importance of working on the factors identified to counteract them and focus care on controlling these risks.

### 4.5 Limitations of the study

Regarding the limitations of the study, it is important to highlight that although the predictive model for mortality managed to obtain a high percentage of specificity, its sensitivity was relatively low. Therefore, it is crucial to continue adjusting this model, incorporating other variables that may improve its predictive capacity. Furthermore, it would be beneficial to apply the model in a multicenter sample to evaluate its performance in different clinical settings since the differences observed between the two cohorts could have affected the generalizability of the results. However, despite these limitations, the model proved to be a valid method to identify patients with a lower risk of mortality.

## 5 Conclusion

Admission to the ICU is frequently associated with chronic noncommunicable pathologies, such as circulatory diseases, respiratory diseases, and cancer, as well as with a higher proportion of older adult patients, whose functional deterioration due to aging and the increasing prevalence of chronic diseases may contribute to this phenomenon. Furthermore, a high percentage of overweight and obese patients was observed in both cohorts.

Our study has the statistical capacity to detect clinically relevant differences in the mortality of patients in our ICU in Cali, independently of the pathologies that motivated their admission. The assessment of nutritional status was carried out by nutrition and dietetics professionals of the hospital and complemented with laboratory data that provided valuable information on the clinical, hematological, and immunological status of the patients upon admission to the ICU.

The development of a predictive mortality model revealed that implementing the MUST screening method and specific nutritional interventions resulted in a significant improvement in the mortality figures of ICU patients. These results highlight the importance of using standardized and validated tools to assess nutritional risk, which can lead to a tangible improvement in the overall health status of patients and a reduction in complications during their ICU stay. This improvement in nutritional care can, in turn, promote faster recovery and better outcomes at hospital discharge.

## Data Availability

The data analyzed in this study is subject to the following licenses/restrictions: the data are not publicly available, due to ethical reasons indicated by the research committee of the health institution, regarding the handling and privacy of patient data. Requests to access these datasets should be directed to Blanca Cecilia Díaz Chavarro, blanca.diaz00@usc.edu.co.
