# Supplementary material for: Impact of nutritional screening on mortality and intensive care unit length of stay
Source: Front Nutr. 2025 Feb 11;12:1474039. doi: 10.3389/fnut.2025.1474039 (PMC11866484; doi:10.3389/fnut.2025.1474039)
Supplement: Supplementary file 1 [file Table_1.docx]

Supplementary Material

# Supplementary Table

| **Table 1.** Hematological variables and their measurement | | |
| --- | --- | --- |
| **Hematological parameter** | | |
| Hemoglobin | Normal range | 12.3–15.3 g/dL |
|  | Anemia | <12.3 g/dL |
|  | Polycythemia | >15.3 g/dL |
| Leukocyte levels | Normal range | 4.5–11 × 10^3^/uL |
|  | Leukocytosis | >11 × 10^3^/uL |
|  | Leukopenia | <4.5 × 10^3^/uL |
| Lymphocyte levels | Normal range | 1.0– 4.8 × 10^3^/uL |
|  | Lymphocytosis | >4.8 × 10^3^/uL |
|  | Lymphopenia | <1.0 × 10^3^/uL |

Abbreviations: g, gram; dL, deciliter; uL, units per liter.
